# Horizontal Gene Transfer of Triazole Resistance in Aspergillus fumigatus

**DOI:** 10.1128/spectrum.01112-22

**Published:** 2022-06-02

**Authors:** Alma Morogovsky, Mariana Handelman, Ammar Abou Kandil, Yona Shadkchan, Nir Osherov

**Affiliations:** a Department of Clinical Microbiology and Immunology, Sackler School of Medicine, Tel-Aviv University, Tel-Aviv, Israel; Universidade de Sao Paulo

**Keywords:** *Aspergillus fumigatus*, horizontal gene transfer, triazole antifungals, drug resistance, hyphal fusion, antifungal resistance

## Abstract

Aspergillus fumigatus is the primary mold pathogen in humans. It can cause a wide range of diseases in humans, with high mortality rates in immunocompromised patients. The first-line treatments for invasive A. fumigatus infections are the triazole antifungals that inhibit Cyp51 lanosterol demethylase activity, blocking ergosterol biosynthesis. However, triazole-resistant strains of A. fumigatus are increasingly encountered, leading to increased mortality. The most common triazole resistance mechanisms in A. fumigatus are alterations in the *cyp51A* gene or promoter. We tested the hypothesis that A. fumigatus can acquire triazole resistance by horizontal gene transfer (HGT) of resistance-conferring gene *cyp51A*. HGT has not been experimentally analyzed in filamentous fungi. Therefore, we developed an HGT assay containing donor A. fumigatus strains carrying resistance-conferring mutated *cyp51A,* either in its chromosomal locus or in a self-replicating plasmid, and recipient strains that were hygromycin resistant and triazole sensitive. Donor and recipient A. fumigatus strains were cocultured and transferred to selective conditions, and the recipient strain tested for transferred triazole resistance. We found that chromosomal transfer of triazole resistance required selection under both voriconazole and hygromycin, resulting in diploid formation. Notably, plasmid-mediated transfer was also activated by voriconazole or hypoxic stress alone, suggesting a possible route to HGT of antifungal resistance in A. fumigatus, both in the environment and during host infection. This study provides, for the first time, preliminary experimental evidence for HGT mediating antifungal resistance in a pathogenic fungus.

**IMPORTANCE** It is well known that bacteria can transfer antibiotic resistance from one strain to another by horizontal gene transfer (HGT), leading to the current worldwide crisis of rapidly emerging antibiotic-resistant bacteria. However, in fungi, HGT events have only been indirectly documented by whole-genome sequencing. This study directly examined fungal HGT of antibiotic resistance in a laboratory setting. We show that HGT of antifungal triazole resistance occurs in the important human fungal pathogen Aspergillus fumigatus. Importantly, we show a plasmid-mediated transfer of triazole resistance occurs under conditions likely to prevail in the environment and in infected patients. This study provides an experimental foundation for future work identifying the drivers and mechanistic underpinnings of HGT in fungi.

## INTRODUCTION

Horizontal gene transfer (HGT), also called “lateral gene transfer,” is the movement of DNA from one organism to another that is not acquired through vertical inheritance (1). HGT can occur between similar organisms or across domains of life.

HGT is well-established and widespread in prokaryotes, most notably involving the transfer of genes participating in metabolism, pathogenicity, and antibiotic resistance. Prokaryotic HGT primarily occurs through conjugation (DNA transfer via cell-cell contact), transformation (cellular uptake of exogenous DNA), and transduction (virus-mediated DNA transfer) (2). Transferred DNA is generally in the form of linear or circular plasmids (self-replicating or integrating), transposons, and bacteriophages.

HGT is also emerging as a small but noticeable contributor to eukaryotic genomes and in particular microbial eukaryotes, such as phytoplankton, extremophiles, parasites, and fungi (1).

In fungi, most HGT events apparently involve bacterial donors: for example, acquisition of bacterial glycosyl hydrolases by rumen fungi and incorporation of a bacterial proline racemase and phenazine superfamily gene, *PhzF*, by Candida parapsilosis (3). Two of the six genes (*BIO3* and *BIO4*) of the Saccharomyces cerevisiae biotin biosynthetic pathway are of bacterial origin (4). Transfer of genetic material between filamentous fungi was also reported: in particular, the apparent transfer of secondary metabolite gene clusters, including all 23 genes of the sterigmatocystin (toxic secondary metabolite) pathway transferred from Aspergillus nidulans to Podospora anserina (5), three genes of the *ACE1* secondary metabolite cluster transferred from Magnaporthe grisea to Aspergillus clavatus (6), and the fuminosin secondary metabolite cluster, possibly transferred from a *Sordariomycete* species to Aspergillus niger (7). The mechanisms of HGT in fungi are not clearly understood but may involve transfer by mycoviruses (8), plasmids (9), transposons (10), and cell fusion followed by parasexual recombination (11–14). Fungal HGT events, rather than being directly studied in a laboratory setting, are indirectly documented by whole-genome sequencing and identification of genes that are phylogenetic outliers originating from the genomes of other organisms (3, 5–7, 9).

Here, we experimentally tested if A. fumigatus, the primary invasive mold pathogen in humans, can undergo chromosomal or plasmid-mediated HGT of voriconazole (VCZ) resistance. VCZ, a triazole antifungal, inhibits fungal Erg11/Cyp51 activity and subsequent ergosterol biosynthesis and is the first-line therapy for invasive aspergillosis (15). However, A. fumigatus is rapidly developing triazole resistance, resulting in increased treatment failure and mortality (16). The most common triazole resistance mechanism in A. fumigatus involves mutations in the *cyp51A* gene or promoter (15).

Previous studies indicate that HGT by cell fusion and diploid formation via the parasexual cycle rarely occur in A. fumigatus under standard laboratory growth conditions but can be forced by mixing auxotrophic haploid strains with different nutritional deficiencies under complementing selection on agar (12). Notably, cell fusion can be induced by selecting prototrophic haploid strains with the triazole antifungal itraconazole, but whether this results in HGT was not tested (14). A. fumigatus can apparently undergo parasexual cycling in patients with chronic Aspergillus diseases, where it was shown that patient biopsy specimens yielded heterokaryons that segregated into different homokaryons (17).

In this study, we tested the hypothesis that a range of extracellular stressors found in the saprophytic or host-infected environments of A. fumigatus activate hyphal fusion and HGT of *cyp51A*(TR46 Y121F T289A) including its TR46 promoter, termed here *cyp51A*^R^, which confers triazole resistance. Our most important findings are that both plasmid-mediated and chromosomal HGT of VCZ resistance can be induced in A. fumigatus. Notably, plasmid-mediated HGT of *cyp51A^R^* is activated by VCZ or hypoxic stress alone, pointing to a possible route for acquiring antifungal resistance by A. fumigatus during host infection.

## RESULTS

### Design and setup of A. fumigatus HGT.

**FIG 1:**
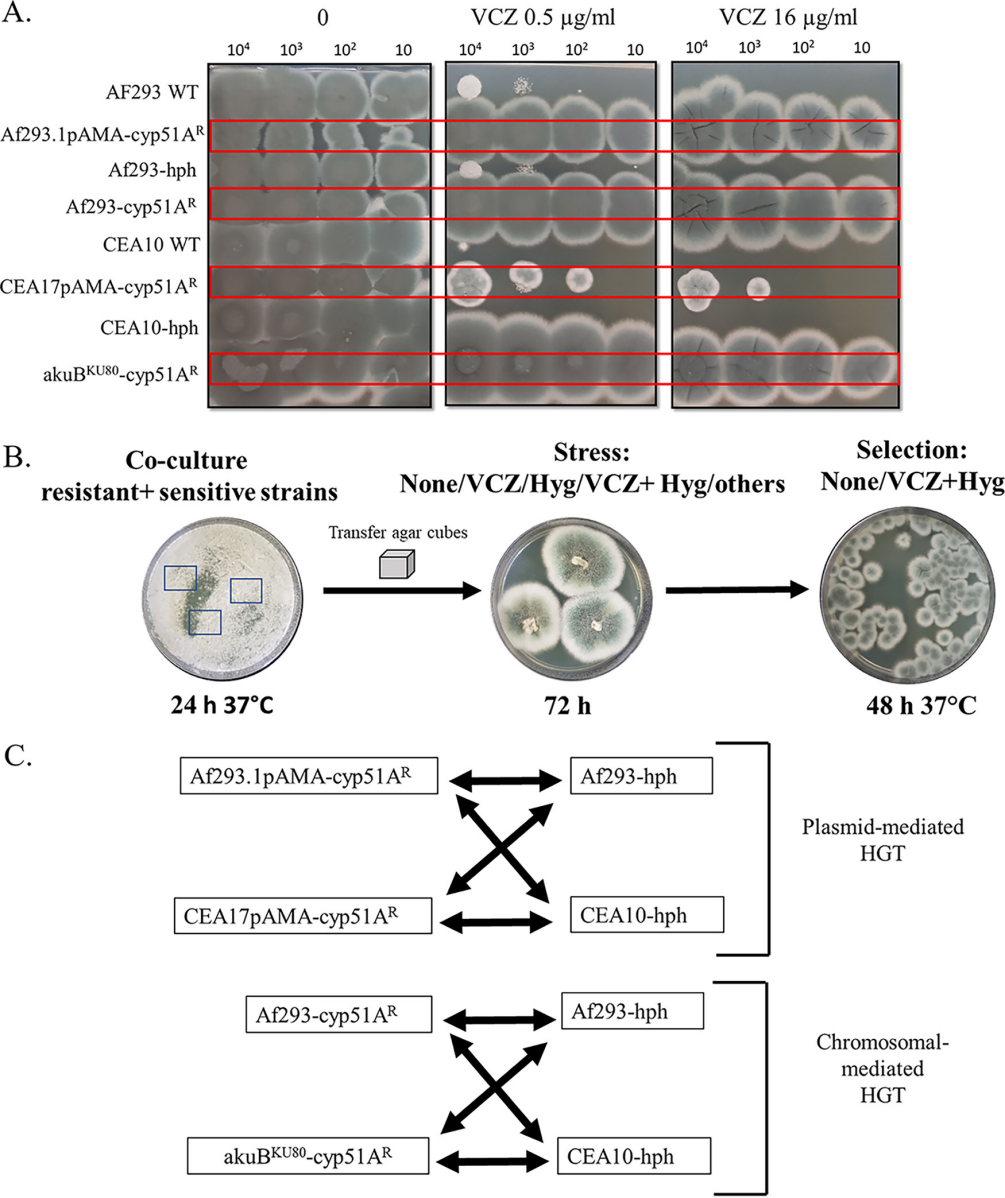
Design and setup of A. fumigatus HGT. (A) Point inoculation of the strains used in this study on YAG agar plates containing increasing concentrations of VCZ. Each droplet column (from left to right) contained 10^4^, 10^3^, 10^2^, or 10 spores per 10-μL droplet. (B) Experimental design of HGT on YAG agar plates. Equal numbers of conidia from a VCZ-resistant strain (donor) and Hyg-resistant (recipient) strain were mixed and plated for 24 h at 37°C, and then agar cubes were excised from the coculture and plated under no/single/double selection for 72 h at 37°C. Conidia were collected and plated under no selection/double selection for 48 h at 37°C. The result of a positive HGT under VCZ+Hyg selection is displayed. (C) Experimental scheme of the HGT experiments carried out in this study. Both plasmid-mediated and chromosomal HGT within and between each genetic background in the combinations outlined were performed.

A. fumigatus lab and environmental strains are haploid, generating unstable transient diploids only during the parasexual and sexual cycles. We tested the ability of A. fumigatus strains to undergo HGT by either plasmid-mediated or chromosomal transfer using two well-characterized but genetically distant haploid strains isolated from infected patients: Af293 and CEA10/CEA17 (18). For plasmid-mediated HGT, we generated triazole-resistant donor strains Af293.1/pAMA-cyp51A^R^ and CEA17/pAMA-cyp51A^R^. These strains contain the self-replicating, high-copy-number, nucleus-localized AMA-1 plasmid (pAMA) enclosing *cyp51A*^R^(Y121F T289A G448S) and TR46-containing promoter (termed *cyp51A*^R^), that confers triazole resistance (19). Importantly, this plasmid does not integrate into the genome or replace the endogenous *cyp51A* gene. For chromosomal HGT, we generated triazole-resistant donor strains Af293-cyp51A^R^ and akuB^KU80^-cyp51A^R^, in which wild-type (WT) *cyp51A* was replaced by targeted integration with *cyp51A*(TR46 Y121F T289A), which confers triazole resistance (20). Triazole-sensitive hygromycin (Hyg)-resistant recipient strains, Af293-hph and CEA10-hph, were generated by targeted insertion of the *hph* hygromycin resistance cassette into the neutral SH1 domain (21). Full details of strain construction and verification are provided in Tables S1 to S3 and Fig. S1 to S3 in the supplemental material. VCZ was tested by broth microdilution and point inoculation on YAG agar plates (Fig. 1A). By broth microdilution, VCZ resistance (MIC, 64 mg/L) was found in strains Af293.1/pAMA-cyp51A^R^, CEA17/pAMA-cyp51A^R^, Af293-cyp51A^R^, and akuB^KU80^-cyp51A^R^. Normal susceptibility (MIC, 0.25 to 0.5 mg/L) was found in the background strains AF293 and CEA10 and the recipient strains Af293-hph and CEA10-hph (Table S4). Point inoculation verified these results (Fig. 1A).

We followed a scheme similar to that used for heterokaryon construction in Aspergillus nidulans test for HGT (22) (Fig. 1B). Equal numbers of conidia from the triazole-resistant donor and -sensitive recipient strains described above were mixed and plated onto YAG agar plates and grown for 24 h at 37°C. One-centimeter agar cubes were then excised and placed on selection/stress plates (containing neither VCZ nor Hyg [“none”], VCZ or Hyg alone, or both) for 72 h at 37C to allow heterokaryon formation by hyphal fusion. To analyze if HGT had occurred, we collected conidia from on and around the agar cubes and allowed them to germinate on selection plates (none or VCZ+Hyg) for 48 h at 37°C (Fig. 1B). Growth on VCZ+Hyg would indicate that the recipient strain (Af293-hph or CEA10-hph) had acquired triazole resistance by HGT. We tested both plasmid-mediated and chromosomal HGT within and between each genetic background in the combinations outlined in Fig. 1C.

### Chromosomal HGT of VCZ resistance in A. fumigatus.

**FIG 2:**
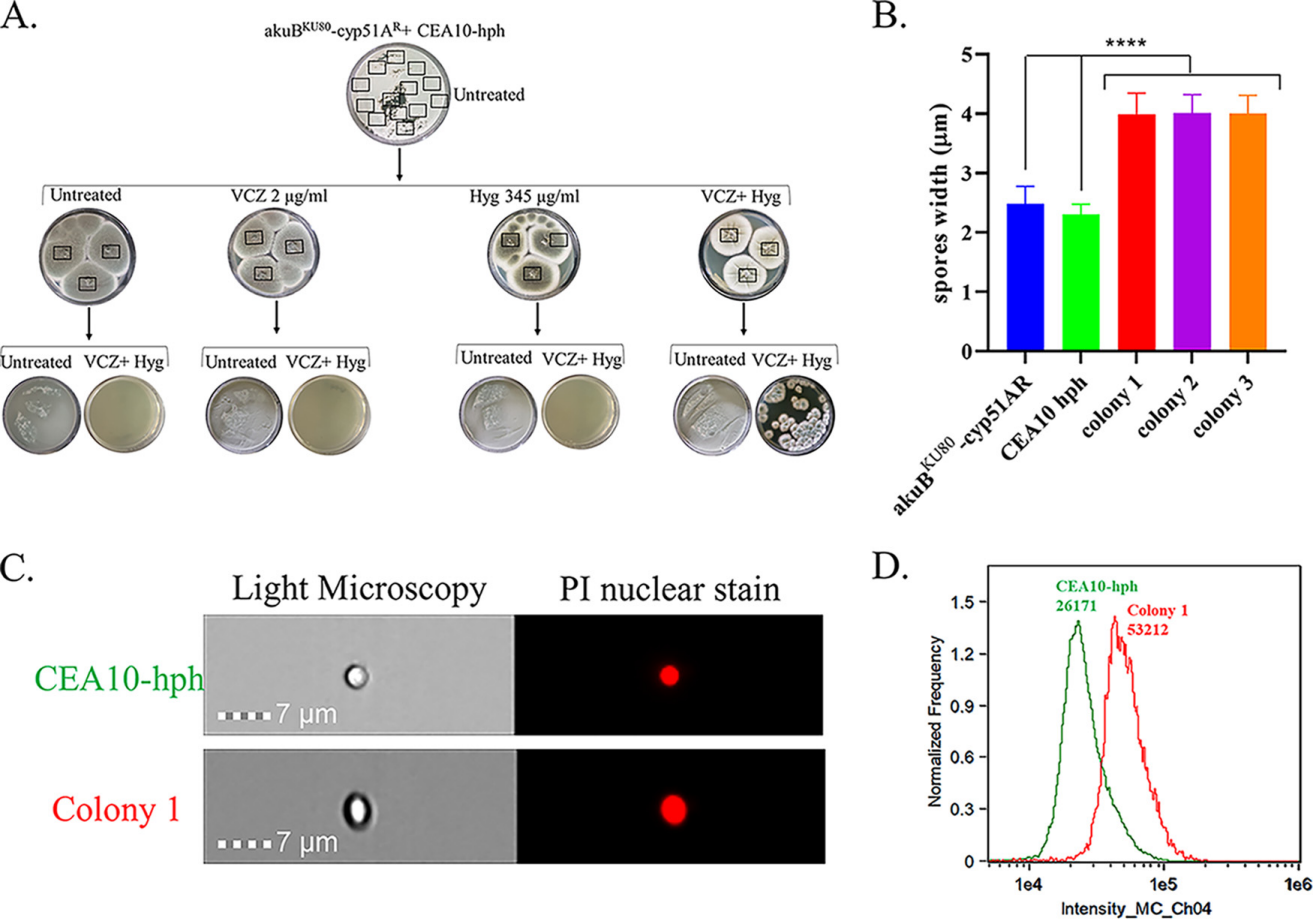
HGT of chromosomal *cyp51A*^R^ in the A. fumigatus CEA10 background. (A) Experimental outline and result demonstrating HGT between akuB^KU80^-cyp51A^R^ donor and CEA10-hph recipient strains under VCZ+Hyg selection. (B) Conidial diameter of akuB^KU80^-cyp51A^R^ and CEA10-hph parental strains and colonies 1 to 3 generated under VCZ+Hyg selection (****, *P* < 0.001). (C) Propidium iodide nuclear staining (left; representative image from flow cytometry analysis) and (D) flow cytometry analysis (right) of CEA10-hph parental strain (green line, mean intensity of 26,171 arbitrary units) and colony 1 (red line, mean intensity 53,212 arbitrary units), generated under VCZ+Hyg selection.

We first tested chromosomal HGT of VCZ resistance because previous studies had determined that diploid formation and gene exchange via parasexual genetics can be forced by mixing auxotrophic strains with different nutritional deficiencies under complementing selection on agar (12). Furthermore, recent work demonstrating that cell fusion between fluorescently tagged germlings is induced by itraconazole or nitrogen starvation (14) led us to hypothesize that we could induce hyphal fusion and subsequent chromosomal HGT of triazole resistance with a single stressor. Chromosomal HGT assays were tested between the strains outlined in Fig. 1C, lower panel. We examined double selection with VCZ (2 μg/mL) plus Hyg (345 μg/mL), and the following single stressors: VCZ (2 μg/mL), Hyg (345 μ/mL), itraconazole (1 to 2 μg/mL), amphotericin B (0.25 μ/mL), caspofungin (0.062 μg/mL), menadione (25 μM), nitrogen starvation, hypoxia (1% O_2_), high temperature (50°C), starvation (agar alone), and acidity (pH 4.0). HGT of VCZ resistance only occurred between the isogenic strains akuB^KU80^-cyp51A^R^ and CEA10-hph under VCZ+Hyg selection and not under any of the single stressors listed above (Fig. 2A). Double VCZ+Hyg selection resulted in 45 ± 16 colonies/10^6^ conidia collected from a single block of agar (*n* = 3 independent experiments) displaying both VCZ and Hyg resistance. Further analysis of three of these colonies confirmed that they were diploid: the conidia they produced were significantly larger (Fig. 2B) and contained double the DNA content, as measured by flow cytometry (Fig. 2C and D). In summary, we show that chromosomal HGT of VCZ resistance occurs between isogenic A. fumigatus strains of the CEA10 background and not the Af293 background through diploid formation but requires strong double selection, as previously described (12).

### Plasmid-mediated HGT of VCZ resistance occurs between A. fumigatus isogenic strains and can occur under VCZ selection alone.

**FIG 3:**
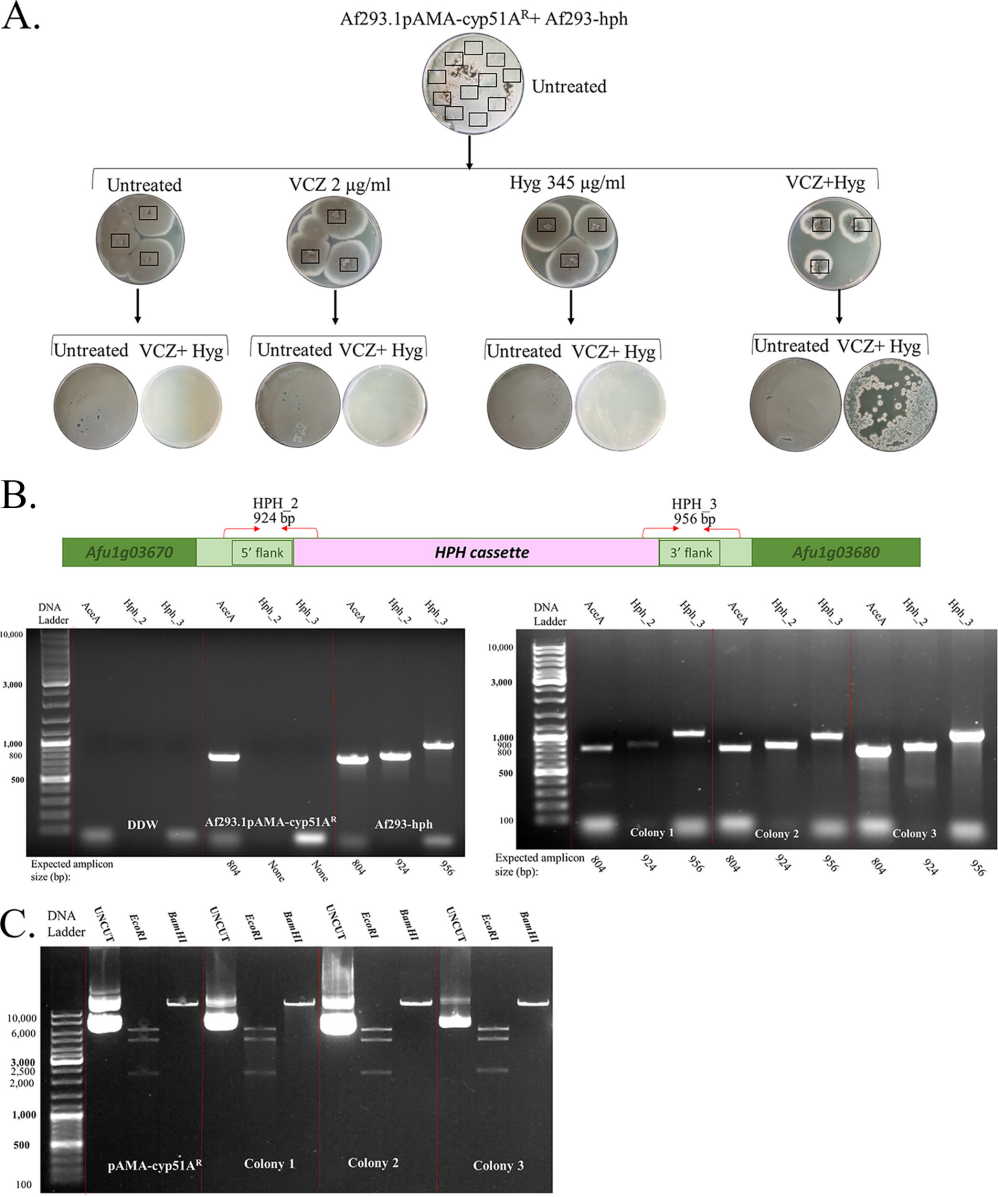
HGT of the *cyp51A*^R^ plasmid in the A. fumigatus Af293 background. (A) Experimental outline and result demonstrating HGT between Af293.1/pAMA-cyp51A^R^ donor and Af293-hph recipient strains under VCZ+Hyg selection. (B) PCR analysis of three colonies (1 to 3) growing under VCZ+Hyg selection after HGT with hph-SH1-specific primer pairs HPH_2 and HPH_3 confirms that they are in the Af293-hph recipient strain background. (C) Plasmid rescue and restriction analysis confirm that recipient colonies 1 to 3 contain pAMA-cyp51A^R^ transferred from the Af293.1/pAMA-cyp51A^R^ donor.

**FIG 4:**
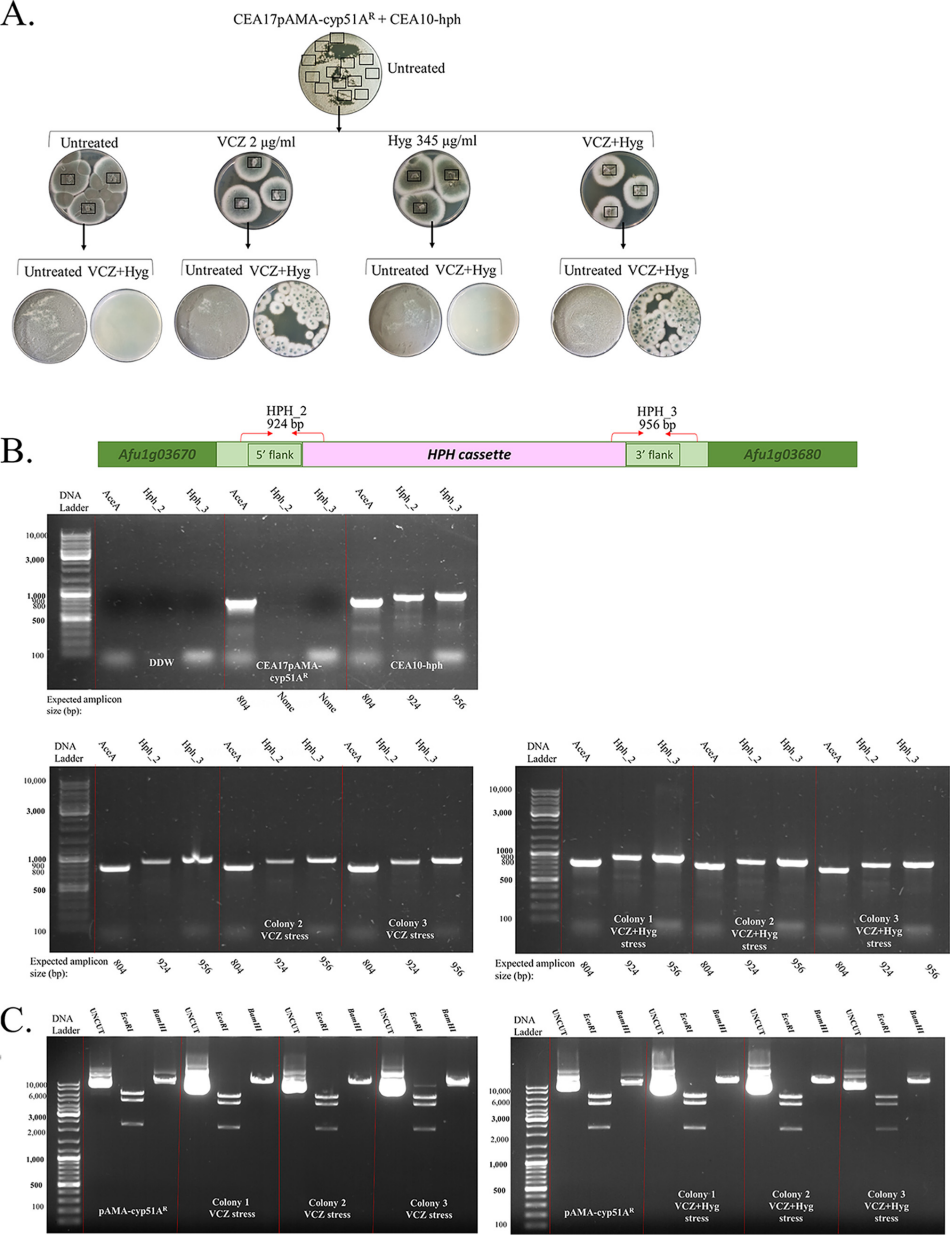
HGT of the *cyp51A*^R^ plasmid in the A. fumigatus CEA10 background under VCZ stress alone. (A) Experimental outline and result demonstrating HGT between CEA17/pAMA-cyp51A^R^ donor and CEA10-hph recipient strains under either VCZ or VCZ+Hyg selection. (B) PCR analysis of three VCZ selection colonies (1 to 3) and three VCZ+Hyg selection colonies (4 to 6) with hph-SH1-specific primer pairs HPH_2 and HPH_3 confirms that they are in the CEA10-hph recipient strain background. (C) Plasmid rescue and restriction analysis confirm that the recipient colonies contain pAMA-cyp51A^R^ transferred from the CEA17/pAMA-cyp51A^R^ donor.

Plasmid-mediated HGT assays were performed between the strains outlined in Fig. 1C (top panel). HGT of VCZ resistance occurred between the Af293.1/pAMA-cyp51A^R^ donor and Af293-hph recipient strain under VCZ+Hyg selection (Fig. 3A), resulting in 70 ± 39 colonies/10^6^ conidia plated (*n* = 3 independent experiments) displaying both VCZ and Hyg resistance. Three colonies were analyzed by PCR and shown to contain the recipient Af293-hph strain-specific locus for Hyg resistance in the SH1 domain, verifying that these colonies were the result of HGT through plasmid pAMA-cyp51A^R^ uptake and expression in the Af293-hph recipient strain, (Fig. 3B). We also isolated the pAMA plasmids from the three recipient colonies and verified by restriction analysis that they were identical to pAMA-cyp51A^R^ in the donor strain (Fig. 3C). Similar experiments between donor CEA17/pAMA-cyp51A^R^ and recipient CEA10-hph strains revealed that HGT took place under VCZ+Hyg selection as described above (234 ± 63 colonies/10^6^ conidia plated), and more excitingly, under VCZ selection alone (108 ± 52 colonies/10^6^ conidia plated) (*n* = 3 independent experiments), as might occur, conceivably, outside a laboratory setting (Fig. 4A). As described above, we verified that these colonies contained the recipient CEA10-hph strain-specific locus (Fig. 4B) and the donor pAMA-cyp51A^R^ plasmid in the recipient strain (Fig. 4C). No HGT was observed between Af293.1/pAMA-cyp51A^R^ and CEA10-hph or CEA17/pAMA-cyp51A^R^ and Af293-hph (data not shown).

### Plasmid-mediated HGT of VCZ resistance occurs in A. fumigatus under hypoxia.

**FIG 5:**
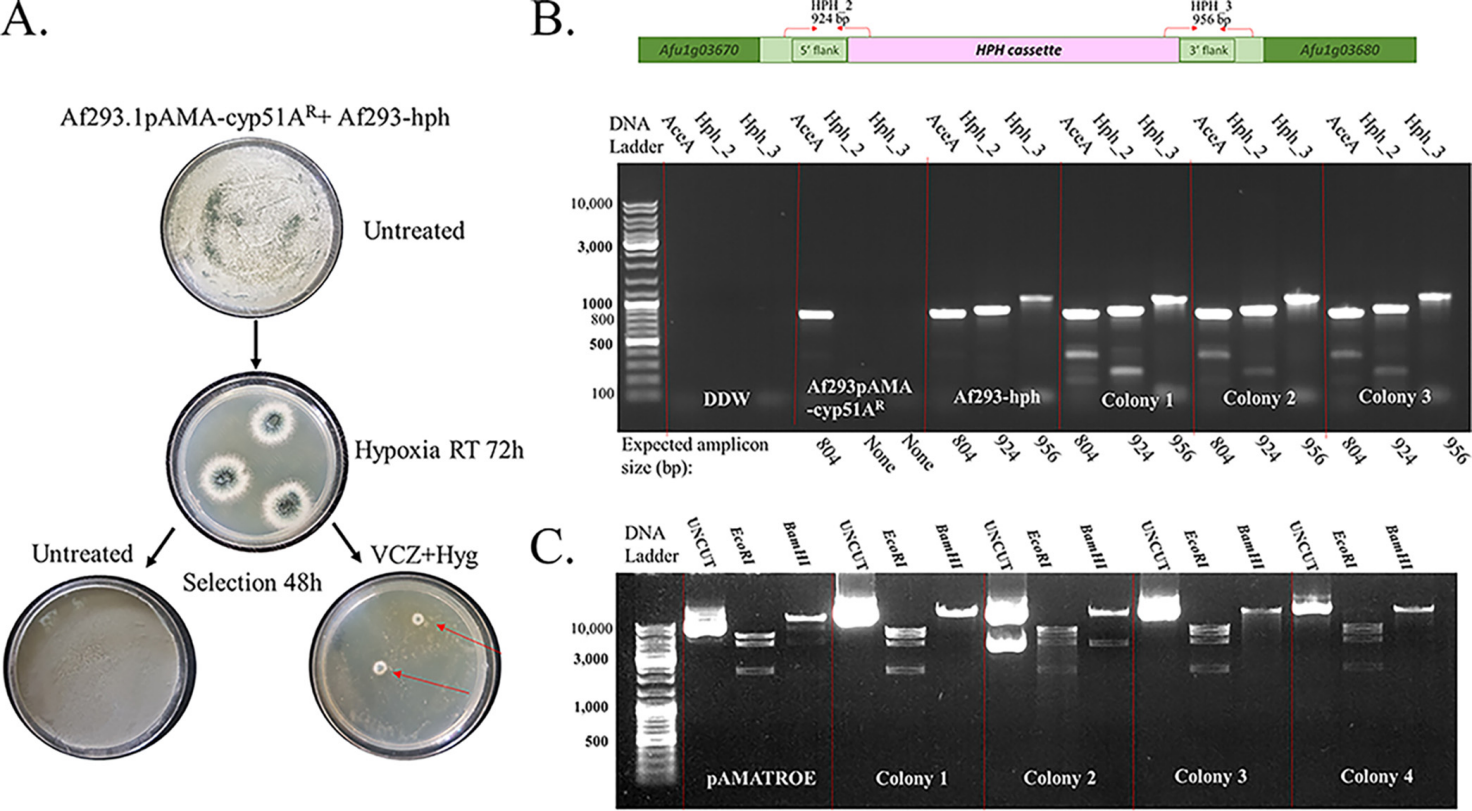
HGT of the *cyp51A*^R^ plasmid in the A. fumigatus Af293 background under hypoxic stress alone. (A) Experimental outline and result demonstrating HGT between Af293.1/pAMA-cyp51A^R^ donor and Af293-hph recipient strains under hypoxic selection. (B) PCR analysis of three colonies (1 to 3) under VCZ+Hyg selection after HGT with hph-SH1-specific primer pairs HPH_2 and HPH_3 confirms that they are in the Af293-hph recipient strain background. (C) Plasmid rescue and restriction analysis confirm that recipient colonies 1 to 3 contain pAMA-cyp51A^R^ transferred from the Af293.1/pAMA-cyp51A^R^ donor.

We tested if various single stressors, including itraconazole (2 μg/mL), amphotericin B (0.25 μg/mL), caspofungin (0.062 μg/mL), menadione (6.12 to 25 μM), nitrogen starvation, hypoxia (1% O_2_), high temperature (50°C), acidity (pH 4.0), and starvation (agar medium) induce plasmid-mediated HGT of VCZ resistance on their own. These experiments were similar to those described above. Results showed that HGT of VCZ resistance occurred under hypoxia between the Af293.1/pAMA-cyp51A^R^ donor and Af293-hph recipient strain, albeit at low efficiency—1 to 2 colonies/10^7^ conidia (*n* = 3 independent experiments) (Fig. 5A). PCR analysis confirmed that the resulting VCZ- and Hyg-resistant colonies contained the Af293-hph strain-specific locus (Fig. 5B) and the donor pAMA-cyp51A^R^ plasmid in the recipient strain (Fig. 5C), indicating that hypoxic stress in YAG plates induced the transfer of the pAMA-cyp51A^R^ plasmid from the Af293.1/pAMA-cyp51A^R^ donor to the Af293-hph recipient strain. Interestingly, HGT was not induced in the CEA10-background strains under any of these stresses, except for VCZ single stress.

## DISCUSSION

As mentioned in the introduction, there is only indirect sequence-based evidence for the existence of HGT in fungi. Direct experimental evidence that can lead to a mechanistic understanding of fungal HGT is lacking. To address this, we set up an experimental system to analyze HGT of triazole resistance in A. fumigatus.

A. fumigatus is the most common invasive mold pathogen in humans and is rapidly developing resistance to triazoles, the front-line drugs for treating invasive aspergillosis (15, 23). We tested for HGT of both chromosomal and plasmid-mediated triazole resistance, using a mutated resistance-conferring allele of *cyp51A*(Y121F T289A G448S) and TR46-containing promoter, encoding lanosterol demethylase, the target of the triazole antifungals. Our experimental system consisted of a haploid donor strain containing either plasmid- or chromosome-located *cyp51A*^R^ and a haploid recipient strain containing the hygromycin selectable marker. We used two isogenic donor/recipient strain pairs, generated in the Af293 and CEA10 backgrounds (18), to test if HGT also occurs between genetically distant A. fumigatus isolates.

We found that chromosomal transfer of triazole resistance occurs only when isogenic donor and recipient haploid strains are placed under double selective pressure (Hyg and VRC, respectively), resulting in diploid formation (Table 1). A. fumigatus diploids are mitotically unstable and can eventually revert to haploids having both selectable markers (22). However, we found that single selective pressure (Hyg/VRC/itraconazole/amphotericin B/caspofungin/heat/oxidative stress/acidity/starvation//hypoxia/nitrogen starvation alone) was insufficient for diploid formation (Table 1). This finding agrees with previous parasexual genetic studies (12, 22) and suggests that chromosomal HGT is unlikely to occur outside a laboratory setting.

**TABLE 1:** Results of HGT experiments performed in this study

Strains	HGT in:			
	VCZ	Hyg	VCZ+Hyg	Hypoxia
Af293.1/pAMA-cyp51A^R^ + Af293-hph	−	−	**+** ^*b*^	**+**
CEA17/pAMA-cyp51A^R^ + CEA10-hph	**+**	−	**+**	−
Af293.1/pAMA-cyp51A^R^ + CEA10-hph	−	−	−	ND^*a*^
CEA17/pAMA-cyp51A^R^ + Af293-hph	−	−	−	ND
Af293-cyp51A^R^ + Af293-hph	−	−	−	ND
akuB^KU80^-cyp51A^R^ + CEA10-hph	−	−	**+**	−
Af293-cyp51A^R^ + CEA10-hph	−	−	−	ND
akuB^KU80^-cyp51A^R^ + Af293-hph	−	−	−	ND

a ND, not determined.

b Significance of bold is to highlight positive ‘+’ results for easier viewing.

To test for plasmid-mediated HGT, we used the AMA-1 based plasmid pAMA-cyp51A^R^. The AMA-1 plasmid, originally isolated from Aspergillus nidulans, is well characterized and widely used (24). It is self-replicating, nonintegrating, nucleus localized, and maintained at approximately 30 copies per nucleus (25). The AMA-1 plasmid is lost upon repeated passaging in the absence of selection (25).

Our most notable result is that plasmid-mediated transfer of triazole resistance does not only occur when donor and recipient strains are placed under Hyg+VRC double selection. Transfer also occurs with single selection under VRC in the CEA10 background or hypoxia in the Af293 background. (Table 1). Sublethal concentrations of VRC and hypoxia can be found in A. fumigatus-infected lungs and could conceivably drive the exchange of mobile resistance elements between infecting strains. Hypoxic conditions also occur within compost heaps where A. fumigatus predominates (26) and in estuarine and hypoxic aquatic environments from which the fungus has been isolated (27). Plasmid-mediated HGT was not observed between the CEA10 and Af293 backgrounds, which suggests that these strains are too genetically distant to exchange the resistance plasmid, most likely due to heterokaryon (het) incompatibility (17). Future experiments will test if HGT occurs between more closely related het-compatible strains of A. fumigatus.

We propose the following model to describe plasmid-mediated HGT in A. fumigatus. Selective stress induces hyphal fusion between donor and recipient strains (14), resulting in nuclear exchange and heterokaryon formation. Nuclear pore disassembly during mitosis of donor nuclei allows plasmid release into the cytosol (28) and entry into mitotically active nuclei of the recipient strain. These nuclei are maintained and replicate under selective pressure. Some eventually form conidia containing both selectable markers, indicating that plasmid-mediated HGT occurred. To confirm this model further, we propose to fluorescently stain the plasmid and image the HGT process between donor and recipient strains by time-lapse fluorescence microscopy (29).

To determine the clinical relevance of the results indicating plasmid-mediated HGT, it will be necessary to better understand naturally occurring plasmids in filamentous fungi. Currently, while some plasmids have been described, most are mitochondrial and contain genes encoding RNA or DNA polymerase. Their function remains unknown (30). Though natural plasmids have not been found in the well-studied fungal genus Aspergillus, no systematic surveys to identify them have been undertaken.

While plasmid-mediated HGT was analyzed here, additional vectors such as transposons (e.g., the Aspergillus impala transposon) or extragenomic particles (e.g., exosomes) need to be examined (31). More stresses can be tested for HGT induction, including copper or iron limitation and excess, nutrient and vitamin limitation, immune cell interaction, bacterial interaction, and *in vivo* stress, by coinfection experiments with the donor/recipient plasma strains in an animal model of infection. This will point to new ways to study HGT in clinical settings.

## MATERIALS AND METHODS

### Media and strains.

A. fumigatus strains were grown on YAG agar plates containing 0.5% yeast extract, 1% dextrose, 10 mM MgSO_4_, vitamin mixture, and trace elements, with 1.5% (wt/vol) agar at pH 7.0 at 37°C. Af293.1 and CEA17, which lack the *pyr4* gene and are thus unable to synthesize uracil and uridine, were grown on YAG UU agar plates (YAG agar medium containing 1% uridine and 1% uracil) for transformation. Where indicated, VCZ and/or hygromycin (Hyg) or other stressors were added to the medium. RPMI-MOPS (morpholinepropanesulfonic acid) (Sigma RPMI cell culture medium with Biological Industries MOPS [0.165 M, pH 7]) liquid medium was used for MIC determination (based on CLSI M38-A2 broth microdilution guidelines [32]). Strains were harvested in 0.02% (vol/vol) Tween 20, resuspended in double-distilled water (DDW), and counted with a hemocytometer. Minimal medium (MM) plates contained 30% NaNO_3_, 1 M KCl, 24.56% MgSO_4_·7H_2_O, 1% dextrose, 1 M KPO_4_, vitamin mixture and trace elements, with 1.5% (wt/vol) agar (pH 7.0).

### Generation of strains.

Generation of the AMA-1-based plasmid pAMA-cyp51A^R^ and transformation into A. fumigatus strains Af293 and CEA17 to generate strains Af293.1/pAMA-cyp51A^R^ and CEA17/pAMA-cyp51A^R^, are described in the supplemental material. Triazole-resistant strains (Af293-cyp51A^R^ or akuB^KU80^-cyp51A^R^) and recipient Hyg-resistant strains (Af293-hph or CEA10-hph) were generated by CRISPR-Cas9-mediated transformation and validation as described in the supplemental material.

### HGT assay and analysis.

A. fumigatus triazole-resistant donor strains (Af293-cyp51A^R^, akuB^KU80^-cyp51A^R^, Af293.1/pAMA-cyp51A^R^, akuB^KU80^, and CEA17/pAMA-cyp51A^R^) and recipient Hyg-resistant strains (Af293-hph or CEA10-hph) were grown on YAG agar for 48 h at 37°C. Conidia were harvested and counted. Conidia (2 × 10^5^) from a triazole-resistant strain and recipient Hyg-resistant strain were mixed and grown for 24 h at 37°C on YAG agar plates. Cubes of agar containing the mixture of growing hyphae from both strains were cut out of these plates and placed on agar plates containing: YAG+VCZ (2 μg/mL VCZ), YAG+Hyg (345 μg/mL Hyg), and YAG+VCZ+Hyg (2 μg/mL VCZ and 345 μg/mL Hyg). We also tested the following stressors for HGT induction on YAG and MM plates: oxidative stress with menadione (6.25 to 25 μM), hypoxic stress with 1% O_2_, high temperature (50°C), nitrogen starvation (MM without nitrate), starvation (agar alone), acidity (pH 4.0), amphotericin B (0.25 μg/mL), caspofungin (0.062 μg/mL), and itraconazole (1 to 2 μg/mL). After 72 h at 37°C or room temperature for hypoxic stress, conidia growing on the cubes or in their vicinity were collected and plated (10^6^ CFU) on YAG/YAG+VCZ+Hyg (2 μg/mL VCZ and 345 μg/mL Hyg) agar plates for 48 h of incubation at 37°C. The colonies that survived on the VCZ+Hyg-supplemented plates were taken as evidence for HGT between the strains and were subsequently passaged, single spore purified under double selection, and tested by colony PCR and restriction analysis.

### Antifungal susceptibility testing: MIC determination.

MICs were obtained following the CLSI M38-A2 broth microdilution guidelines for antifungal susceptibility testing (32). Briefly, the assay was done with an inoculum suspension of 5 × 10^3^ CFU per well in RPMI-MOPS medium. The MIC was determined after 48 h of incubation at 37°C. (The MIC was determined from the first well that displayed complete growth inhibition by microscopy).

### Droplet growth inhibition assay.

Freshly harvested conidia were serially diluted in sterile double-distilled water (SDDW) plus 0.02% Tween to obtain defined concentrations of 10^6^, 10^5^, 10^4^, and 10^3^ CFU/mL. Conidia were spotted in a volume of 10 μL on YAG plates under the specified stress-inducing agents. Growth was documented after 48 h of incubation at 37°C.

### Multispectral imaging flow cytometry (ImageStreamX) analysis.

Conidia were fixed and stained with propidium iodide (PI) and imaged by multispectral imaging flow cytometry (ImageStreamX mark II; Amnis Corp., Seattle, WA). The laser setting was 561 nm = 7.5 mW. At least 10^4^ focused images of single-stained conidia were collected from each sample. Data were analyzed with the manufacturer’s image analysis software (IDEAS 6.2; Amnis Corp.). Cells were gated for single cells using the area and aspect ratio features and for focused cells by the Gradient RMS feature. The intensity of nuclear content stained with PI was analyzed.

## References

[1] Van Etten J, Bhattacharya D. 2020. Horizontal gene transfer in eukaryotes: not if, but how much? Trends Genet 36:915–925. doi:10.1016/j.tig.2020.08.006. 33012528

[2] von Wintersdorff CJ, Penders J, van Niekerk JM, Mills ND, Majumder S, van Alphen LB, Savelkoul PH, Wolffs PF. 2016. Dissemination of antimicrobial resistance in microbial ecosystems through horizontal gene transfer. Front Microbiol 7:173. doi:10.3389/fmicb.2016.00173. 26925045 PMC4759269

[3] Fitzpatrick DA. 2012. Horizontal gene transfer in fungi. FEMS Microbiol Lett 329:1–8. doi:10.1111/j.1574-6968.2011.02465.x. 22112233

[4] Hall C, Dietrich FS. 2007. The reacquisition of biotin prototrophy in *Saccharomyces cerevisiae* involved horizontal gene transfer, gene duplication and gene clustering. Genetics 177:2293–2307. doi:10.1534/genetics.107.074963. 18073433 PMC2219469

[5] Slot JC, Rokas A. 2011. Horizontal transfer of a large and highly toxic secondary metabolic gene cluster between fungi. Curr Biol 21:134–139. doi:10.1016/j.cub.2010.12.020. 21194949

[6] Khaldi N, Collemare J, Lebrun MH, Wolfe KH. 2008. Evidence for horizontal transfer of a secondary metabolite gene cluster between fungi. Genome Biol 9:R18. doi:10.1186/gb-2008-9-1-r18. 18218086 PMC2395248

[7] Khaldi N, Wolfe KH. 2011. Evolutionary origins of the fumonisin secondary metabolite gene cluster in *Fusarium verticillioides* and *Aspergillus niger*. Int J Evol Biol 2011:423821. doi:10.4061/2011/423821. 21716743 PMC3119522

[8] Kotta-Loizou I, Coutts RHA. 2017. Mycoviruses in aspergilli: a comprehensive review. Front Microbiol 8:1699. doi:10.3389/fmicb.2017.01699. 28932216 PMC5592211

[9] Rosewich UL, Kistler HC. 2000. Role of horizontal gene transfer in the evolution of fungi. Annu Rev Phytopathol 38:325–363. doi:10.1146/annurev.phyto.38.1.325. 11701846

[10] Palmer JM, Keller NP. 2010. Secondary metabolism in fungi: does chromosomal location matter? Curr Opin Microbiol 13:431–436. doi:10.1016/j.mib.2010.04.008. 20627806 PMC2922032

[11] Engel T, Verweij PE, van den Heuvel J, Wangmo D, Zhang J, Debets AJM, Snelders E. 2020. Parasexual recombination enables *Aspergillus fumigatus* to persist in cystic fibrosis. ERJ Open Res 6:00020-2020. doi:10.1183/23120541.00020-2020. 33313304 PMC7720686

[12] Firon A, Beauvais A, Latge JP, Couve E, Grosjean-Cournoyer MC, D'Enfert 0.029w?>C. 2002. Characterization of essential genes by parasexual genetics in the human fungal pathogen *Aspergillus fumigatus*: impact of genomic rearrangements associated with electroporation of DNA. Genetics 161:1077–1087. doi:10.1093/genetics/161.3.1077. 12136012 PMC1462181

[13] Pontecorvo G, Roper JA, Forbes E. 1953. Genetic recombination without sexual reproduction in Aspergillus niger. J Gen Microbiol 8:198–210. doi:10.1099/00221287-8-1-198. 13035047

[14] Macdonald D, Thomson DD, Johns A, Contreras Valenzuela A, Gilsenan JM, Lord KM, Bowyer P, Denning DW, Read ND, Bromley MJ. 2019. Inducible cell fusion permits use of competitive fitness profiling in the human pathogenic fungus *Aspergillus fumigatus*. Antimicrob Agents Chemother 63:e01615-18. doi:10.1128/AAC.01615-18. 30397071 PMC6325235

[15] Garcia-Rubio R, Cuenca-Estrella M, Mellado E. 2017. Triazole resistance in Aspergillus species: an emerging problem. Drugs 77:599–613. doi:10.1007/s40265-017-0714-4. 28236169

[16] Verweij PE, Chowdhary A, Melchers WJ, Meis JF. 2016. Azole resistance in *Aspergillus fumigatus*: can we retain the clinical use of mold-active antifungal azoles? Clin Infect Dis 62:362–368. doi:10.1093/cid/civ885. 26486705 PMC4706635

[17] Zhang J, Snelders EE, Zwaan BJ, Schoustra SE, Kuijper EJ, Arendrup MC, Melchers WJG, Verweij PE, Debets AJM. 2019. Relevance of heterokaryosis for adaptation and azole-resistance development in *Aspergillus fumigatus*. Proc Biol Sci 286:20182886. doi:10.1098/rspb.2018.2886. 30963936 PMC6408600

[18] Bertuzzi M, van Rhijn N, Krappmann S, Bowyer P, Bromley MJ, Bignell EM. 2021. On the lineage of *Aspergillus fumigatus* isolates in common laboratory use. Med Mycol 59:7–13. doi:10.1093/mmy/myaa075. 32944768 PMC7779236

[19] Osherov N, Kontoyiannis DP, Romans A, May GS. 2001. Resistance to itraconazole in *Aspergillus nidulans* and *Aspergillus fumigatus* is conferred by extra copies of the A. nidulans P-450 14alpha-demethylase gene, *pdmA*. J Antimicrob Chemother 48:75–81. doi:10.1093/jac/48.1.75. 11418514

[20] Wiederhold NP, Gil VG, Gutierrez F, Lindner JR, Albataineh MT, McCarthy DI, Sanders C, Fan H, Fothergill AW, Sutton DA. 2016. First detection of TR34 L98H and TR46 Y121F T289A Cyp51 mutations in *Aspergillus fumigatu*s isolates in the United States. J Clin Microbiol 54:168–171. doi:10.1128/JCM.02478-15. 26491179 PMC4702720

[21] Pham T, Xie X, Lin X. 2020. An intergenic “safe haven” region in *Aspergillus fumigatus*. Med Mycol 58:1178–1186. doi:10.1093/mmy/myaa009. 32171003

[22] Todd RB, Davis MA, Hynes MJ. 2007. Genetic manipulation of *Aspergillus nidulans*: heterokaryons and diploids for dominance, complementation and haploidization analyses. Nat Protoc 2:822–830. doi:10.1038/nprot.2007.113. 17446882

[23] Latge JP, Chamilos G. 2019. *Aspergillus fumigatus* and aspergillosis in 2019. Clin Microbiol Rev 33:e00140-18. doi:10.1128/CMR.00140-18. 31722890 PMC6860006

[24] Aleksenko A, Clutterbuck AJ. 1997. Autonomous plasmid replication in *Aspergillus nidulans*: AMA1 and MATE elements. Fungal Genet Biol 21:373–387. doi:10.1006/fgbi.1997.0980. 9290250

[25] Gems D, Johnstone IL, Clutterbuck AJ. 1991. An autonomously replicating plasmid transforms *Aspergillus nidulans* at high frequency. Gene 98:61–67. doi:10.1016/0378-1119(91)90104-j. 2013411

[26] Zhang J, Snelders E, Zwaan BJ, Schoustra SE, Meis JF, van Dijk K, Hagen F, van der Beek MT, Kampinga GA, Zoll J, Melchers WJG, Verweij PE, Debets AJM. 2017. A novel environmental azole resistance mutation in *Aspergillus fumigatus* and a possible role of sexual reproduction in its emergence. mBio 8:e00791-17. doi:10.1128/mBio.00791-17. 28655821 PMC5487732

[27] Lee S, Park MS, Lim YW. 2016. Diversity of marine-derived aspergillus from tidal mudflats and sea sand in Korea. Mycobiology 44:237–247. doi:10.5941/MYCO.2016.44.4.237. 28154481 PMC5287156

[28] De Souza CP, Osmani AH, Hashmi SB, Osmani SA. 2004. Partial nuclear pore complex disassembly during closed mitosis in *Aspergillus nidulans*. Curr Biol 14:1973–1984. doi:10.1016/j.cub.2004.10.050. 15556859

[29] Srinivasan C, Lee J, Papadimitrakopoulos F, Silbart LK, Zhao M, Burgess DJ. 2006. Labeling and intracellular tracking of functionally active plasmid DNA with semiconductor quantum dots. Mol Ther 14:192–201. doi:10.1016/j.ymthe.2006.03.010. 16698322

[30] Griffiths AJ. 1995. Natural plasmids of filamentous fungi. Microbiol Rev 59:673–685. doi:10.1128/mr.59.4.673-685.1995. 8531891 PMC239394

[31] Evangelinos M, Anagnostopoulos G, Karvela-Kalogeraki I, Stathopoulou 0.029w?>PM, Scazzocchio C, Diallinas G. 2015. Minos as a novel Tc1/mariner-type transposable element for functional genomic analysis in *Aspergillus nidulans*. Fungal Genet Biol 81:1–11. doi:10.1016/j.fgb.2015.05.007. 26021704

[32] Clinical and Laboratory Standards Institute. 2008. Reference method for broth dilution antifungal susceptibility testing of filamentous fungi: approved standard, 2nd ed. CLSI document M38-A2. Clinical and Laboratory Standards Institute, Wayne, PA.

