# Supplementary material for: Horizontal Gene Transfer of Triazole Resistance in Aspergillus fumigatus
Source: Microbiol Spectr. 2022 Jun 2;10(3):e01112-22. doi: 10.1128/spectrum.01112-22 (PMC9241940; doi:10.1128/spectrum.01112-22)
Supplement: Supplemental file 1 — Supplemental material. Download spectrum.01112-22-s0001.pdf, PDF file, 0.6 MB [file spectrum.01112-22-s0001.pdf]

Supplementary materials:

Table S1. Strains used in this study

Strains	Genotype	Reference
Af293	Wild type; MAT1-2	Pain et al., 2004 (1)
Af293.1	*pyrG*$^{-}$; MAT1-2	Osherov et al., 2001 (2)
CEA10	Wild type; MAT1-1	Girardin et al., 1993 (3)
CEA17	*pyrG*$^{-}$; MAT1-1	D'Enfert., 1996 (4)
akuB^{KU80}	ΔakuB^{ku80}::*pyrG*^{Af}*-zeo*; MAT1-1	Da Silva Ferreira et al., 2006 (5)
Af293.1pAMA-cyp51A^{R}	Af293.1	This study
Af293-hph	Af293::*SH1-hph*	This study
Af293-cyp51A^{R}	Af293;*Δcyp51A+::cyp51A*^{R}	This study
	TR46\Y121F/T289A/G448S (Azole^{R}*)*	This study
CEA17pAMA-cyp51A^{R}	CEA17	This study
CEA10-hph	CEA10::*SH1-hph*	This study
akuB^{KU80}-cyp51A^{R}	ΔakuB^{ku80}::*pyrG*^{Af}*-zeo;Δcyp51A+::cyp51A*^{R}	
	TR46\Y121F/T289A/G448S (Azole^{R}*)*	
AF1167	Clinical azole resistant patient isolate	RBA*
Af293.1pAMA1	Af293	This study
CEA17 pAMA1	CEA17	This study

*Prof. Ronen Ben-Ami, Sourasky Medical Center, Tel Aviv University, Tel Aviv.

Table S2. Primers used in this study.

**Primer name**	**Sequence (5'-3')**
pAMA-cyp51A^{R} _F	attcgagctcggtacccgggGAATATATACGTCGATCTGTGTGAC
pAMA-cyp51A^{R} _R	cctgcaggtcgactctagagCAGGTTTTCGCACGAGCTT
SH1_hph_pGEM_F	TTCTATTCCCATCCATGGTAGAATAGCCAGTGACTCTGCAAAGTCTACCGcctaggtacagaagtccaattgc
SH1_hph_pGEM_R	CGAAAAAGAAGGGGCAGGAACCTGTTGTGTTCTATTCACAGTCATACATCCGtctagaaagaaggattacctcta
AceA_F	ATGCTCATCGATGGCGAGAA
AceA_R	CGGGCGATCCATGTTCAGAAT
HPH_1_F	GGGAGAAGAAAGCTAAGAACGAA
HPH_1_R	ACGTGGGTACGGCAAGTGAA
HPH_2_F	GGGAGAAGAAAGCTAAGAACGAA
HPH-2_R	AGATCGGAAGCAATTGGAC
HPH_3_F	TGCACAGGTACACTTGTTTAGAGG
HPH_3_R	CATCATGCCTCTGGATATCTATGC
cyp51A^{R}_F	TGGCTGATCAAACTATGCTCATG
cyp51A^{R}_R	TGGTTACAACAGTCTCACTTGGA
cyp51B_F	TGCATTGGCGAGCAATT
cyp51B_R	TCAGGCTTTGGTAGCGGACTC
TR_ F	CTCCTCTAGAAAAAACTCATGAGTG
TR_ R	GTATGGTATGCTGGAACTACACCTT

Table S3. crRNAs used in this study

crRNA name	Use	Sequence
crRNA_hph	crRNA for cutting SH1	GAAGTGATGGCTGGTTGAAG
crRNA1_TR	crRNA for cutting *Cyp51A 5'* side	GGCTTTCATATGTTGCTCAG
crRNA2_TR	crRNA for cutting *Cyp51A 3'* side	GAAGCCAAGCATCATCGGCT

pAMA-cyp51A^R plasmid construction. We isolated genomic DNA from clinical isolate AF1167 (Table S1) harboring *cyp51A^R* (TR46\Y121F/T289A/G448S) with a TR46-containing promoter that confers triazole-resistance. We PCR amplified this gene including 1000 bp 5' and 500 bp 3' to the ORF with primers pAMA-cyp51A^R _F and pAMA-cyp51A^R _R (Table S2) using AF1167 genomic DNA as template. We performed Gibson assembly of the mutant gene into the *BamH*I-linearized pAMA-1 self-replicating nuclear-localized high copy number plasmid used for gene overexpression in *A. fumigatus* (2). The resulting pAMA-cyp51A^R plasmid (Fig. S1) was verified by PCR analysis and sequencing (not shown).

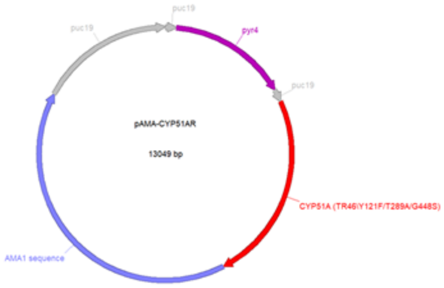

Fig S1. pAMA-cyp51A^R plasmid construct for Af293 and CEA17 transformation. Plasmid AMA1 was linearized with *BamH*I and inserted with *CYP51A^R (*TR46\Y121F/T289A/G448S).

CRISPR-Cas9 mediated transformation of *A. fumigatus*: Transformation was carried out according to Abdallah et al., 2017 (6), with minor modifications. 1x10^6/ml conidia were inoculated into a petri dish containing 30 ml liquid Sabouraud (SAB) medium, wrapped with parafilm and incubated for 16-18 h at 37°C. Mycelia were harvested by filtration with sterilized miracloth, washed with 20 ml SAB, and transferred to 10 ml of freshly filtered protoplasting solution (0.6M KCl, 50 mM $CaCl_2$, 5 mM Tris-HCl (pH 7.5), 5% VinoTaste FCE Novo Nordisk) in a 50 ml tube and incubated for 2 h at 30ºC in a shaker (horizontal position) at 70 rpm until protoplasts were produced. Protoplasts were filtered through sterile miracloth and the flow-through containing the protoplasts was transferred to ice and centrifuged at 3000 rpm for 5 min at RT. The pellet was resuspended in 1 ml protoplast buffer (0.6M KCl, 50 mM $CaCl_2$, 5 mM Tris-HCl (pH 7.5) and transferred to ice. Protoplasts were counted using a hemocytometer and adjusted to 5x10^6 protoplasts/ml with protoplast buffer (on ice).

For CRISPR, the RNP complex was incubated for 5 minutes at RT before use (see Table S3 for CRISPR RNA (crRNA) sequences). Per one plate: 105 µl of protoplast solution, 0.5-1 µg DNA (plasmid) or 0.5-

1 µg DNA template for CRISPR, 3 ml RNP complex and 25 µl PEG buffer (0.6M KCl, 50 mM $CaCl_2$, 5 mM Tris-HCl (pH 7.5), 40% PEG3350) were pipetted gently 3-4 times to mix and then incubated on ice for 25 min (for DNA plasmid transformation) and 50 min (for CRISPR). Then 250 µl of PEG buffer were added, mixed and incubated 1-2 min for plasmid DNA and 20 min for CRISPR at room temperature. The solution was transferred to a 50 ml tube with 6 ml TPGS TOP agar (yeast extract 2%, Peptone 0.5%, D-glucose 2%, Sucrose 1M and Agar 0.7%, titrated to pH 6) supplemented with selection (VCZ 4 µg/ml or Hyg 200 µg/ml) and poured to YPGS selection plates (yeast extract 2%, Peptone 0.5%, D-glucose 2%, Sucrose 1M, Agar 1.5%, titrated to pH 6). Plates were incubated for 1h at RT, then transferred wrapped with parafilm to 37ºC for at least 48 h. Then, transformants were transferred again to YAG+ selection plates by isolation streaking and individual colonies were picked for validation by PCR.

Construction and verification of strains Af293.1pAMA-cyp51A^{R}and CEA17pAMA-cyp51A^R. pAMA-cyp51A^R was transformed into the AF293.1 and CEA17 strains (uridine and uracil auxotrophs) to generate strains Af293.1pAMA-cyp51A^R and CEA17pAMA-cyp51A^R. To verify that the Af293.1pAMA-cyp51A^R and CEA17pAMA-cyp51A^R strains were indeed azole resistant, we tested them by the broth microdilution assay in the presence of voriconazole (Table S4). We tested three independent Af293.1pAMA-cyp51A^R (1,2,3) and CEA17pAMA-cyp51A^R (1,2,3) colonies from each strain, comparing them to Af293 and CEA10 wild type azole sensitive strains. All strains were as azole-resistant as the AF1167 strain from which *CYP51A^R (*TR46\Y121F/T289A/G448S) was cloned.

Table S4. VCZ MICs of strains used in this study.

**Strain**	**VCZ MIC (mg/L)**
Af293	0.25
CEA10	0.50
Af293.1pAMA1	0.50
CEA17 pAMA1	0.25
Af293-hph	0.25
CEA10-hph	0.50
AF1167	>64
Af293.1pAMA-cyp51A^R 1,2,3	>64
CEA17pAMA-cyp51A^R 1,2,3	>64
Af293-cyp51A^R 1,2,3	>64
akuB^{KU80}-cyp51A^R 1,2,3	>64

Construction and verification of strains Af293-hph and CEA10-hph. Primers SH1_hph_pGEM_F and SH1_hph_pGEM_R (Table S2) were used for amplification of the hygromycin resistance (*hph*) cassette, with 23 bp flanking the *SH1* 5' and 3' ends of the region. The 2,454 bp amplicon was introduced into Af293 and CEA10 strains (Table S1), along with the gRNA crRNA_hph for the target region (Table S3) and Cas9 enzyme (IDT). Transformant colonies were screened on YPGS-hygromycin (200 µg/ml) agar plates, after which selected colonies were streaked twice on YAG-hygromycin (350 µg/ml) agar plates. Mutants were verified by PCR with primers set to determine correct integration of the *hph* cassette into the target locus (primers " HPH_1_F " and " HPH_1_R ", " HPH_2_F " and "HPH_2_R" and primers "HPH_3_F " and " HPH_3_R ") (Table S2 and Fig. S2).

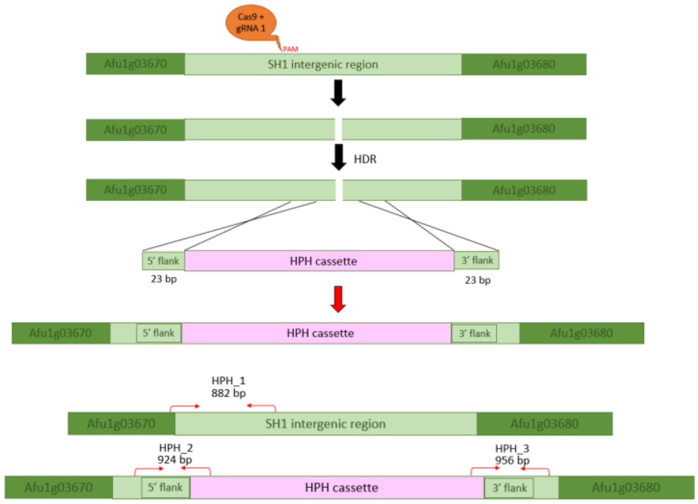

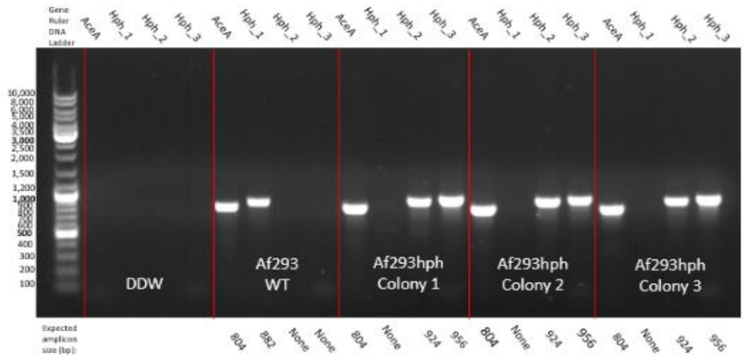

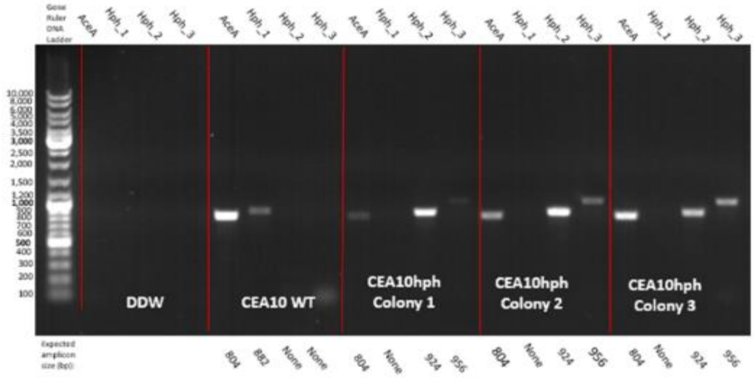

Fig. S2. PCR verification of Af293-hph and CEA10-hph transformants harboring the *hph* cassette. Mutants were verified by PCR with primers set to determine the correct integration of the *hph* cassette into the target locus (top scheme, primers " HPH_2_F" and " HPH-2_R" and primers HPH_3_F and HPH_3_R) for correct integration of the *hph* cassette into the target locus of strains Af293 (top gel) and CEA10 (lower gel). Primers amplifying *aceA* were used as a positive PCR control.

Construction and verification of Af293-cyp51A^R or akuB^{KU80}-cyp51A^R. Primers cyp51AR_F and cyp51AR_R (Table S2) were used for amplification of the CYP51A TR46\Y121F/T289A/G448S gene from the AF1167 strain (Table S1), with 75 bp flanking the gene 5' and 46 bp flanking the gene 3'. The 2,175 bp amplicon was transformed into the *Af293* and *akuB^{KU80}* strains, along with two gRNAs, one for each 5' and 3' ends of the target gene (crRNAs " crRNA1_TR " and “crRNA2_TR”, Table S3), and Cas9 enzyme (IDT). Transformant colonies were screened on YPGS-voriconazole (16 µg/ml) agar plates, after which selected colonies were streaked twice on YAG- voriconazole (16 µg/ml) agar plates. Mutants were verified by PCR with primers set to amplify the promoter region with tandem repeat 46 bp to determine correct integration of the gene into the target locus (primers " TR_ F" and " TR_ R") (Table S2 and Fig S3). We tested three independent Af293-cyp51A^R (1,2,3) and akuB^{KU80}-cyp51A^R (1,2,3) colonies from each strain, comparing them to Af293 and CEA10 wild type azole sensitive strains (Table S4). All strains were as azole-resistant as the AF1167 strain from which *CYP51A^R* (TR46\Y121F/T289A/G448S) was cloned.

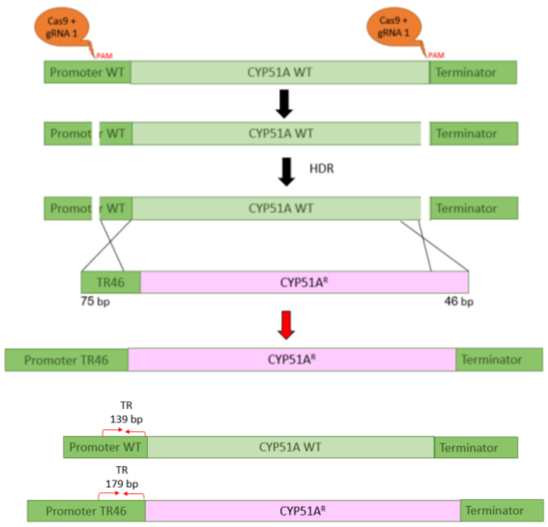

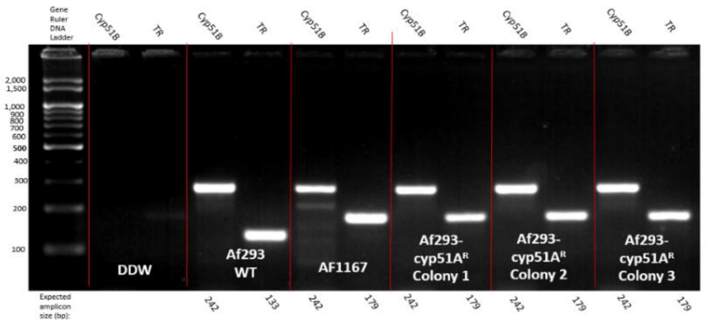

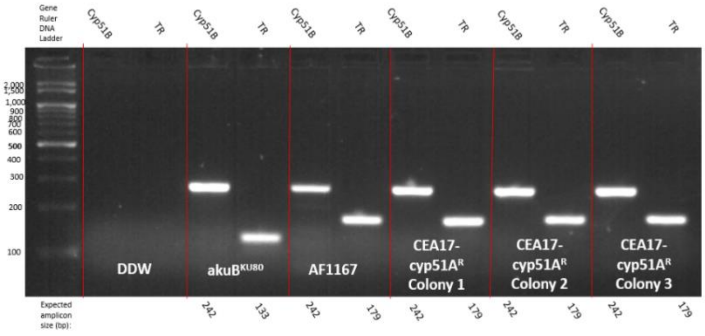

Fig. S3. Generation of Af293-cyp51A^{R} and akuB^{KU80}-cyp51A^{R} transformants harboring cyp51A^{R}. Mutants were verified by PCR with primers set to amplify the promoter region with tandem repeat 46 bp to determine correct integration of the gene into the target locus (primers " TR_ F" and " TR_ R", top scheme). PCR amplicons for three independent transformants of Af293-cyp51A^{R} (left panel) and akuB^{KU80}-cyp51A^{R} (right panel) are shown. Primers cyp51B_F and cyp51B_R amplifying *cyp51B* were used as a positive PCR control. Control strains include Af293, akuBKU80, and AF1167, from which the cyp51A^{R} gene is derived.

References

1. Pain, A., Woodward, J., Quail, M. A., Anderson, M. J., Clark, R., Collins, M., Fosker, N., Fraser, A., Harris, D., Larke, N., Murphy, L., Humphray, S., O'Neil, S., Pertea, M., Price, C., Rabbinowitsch, E., Rajandream, M. A., Salzberg, S., Saunders, D.,Hall, N. (2004). Insight into the genome of *Aspergillus fumigatus*: Analysis of a 922 kb region encompassing the nitrate assimilation gene cluster. Fungal Genetics and Biology, 41(4), 443–453.
2. Osherov, N., Kontoyiannis, D. P., Romans, A., & May, G. S. (2001). Resistance to Itraconazole in *Aspergillus nidulans* and *Aspergillus fumigatus* is conferred by extra copies of the *A. nidulans* P-450 14α-demethylase gene, *pdmA*. Journal of Antimicrobial Chemotherapy, 48(1), 75–81.
3. Girardin, H., Latge, J. P., Srikantha, T., Morrow, B., & Soll, D. R. (1993). Development of DNA probes for fingerprinting *Aspergillus fumigatus*. Journal of Clinical Microbiology, 31(6), 1547–1554.
4. D'Enfert, C. (1996). Selection of multiple disruption events in *Aspergillus fumigatus* using the orotidine-5'-decarboxylase gene, *pyrG*, as a unique transformation marker. Current Genetics, 30(1), 76–82.
5. da Silva Ferreira ME., Kress MR, Savoldi M., et al. (2006) The akuBKU80 mutant deficient for nonhomologous end joining is a powerful tool for analyzing pathogenicity in *Aspergillus fumigatus*. Eukaryot Cell, 5: 207–211.
6. Al Abdallah., Q., Ge., W., & Fortwendel., J. R. (2017). Simple and Universal System for Gene Manipulation in *Aspergillus fumigatus*: In Vitro -Assembled Cas9-Guide RNA Ribonucleoproteins Coupled with Microhomology Repair Templates. MSphere, 2(6), 1–14.
